# ROR1 is a novel prognostic biomarker in patients with lung adenocarcinoma

**DOI:** 10.1038/srep36447

**Published:** 2016-11-10

**Authors:** Yu-Zhu Zheng, Rui Ma, Jian-Kang Zhou, Cheng-Lin Guo, Yong-Sheng Wang, Zheng-Guang Li, Lun-Xu Liu, Yong Peng

**Affiliations:** 1 Department of Thoracic Surgery, State Key Laboratory of Biotherapy/Collaborative Innovation Center for Biotherapy, West China Hospital, Sichuan University, Chengdu, Sichuan Province, P.R. China; 2 The Third People’s Hospital of Chengdu, Chengdu, Sichuan Province, P.R. China

## Abstract

Currently, there is no reliable biomarker to clinically predict the prognosis of lung adenocarcinoma (ADC). The receptor-tyrosine-kinase like orphan receptor 1 (ROR1) is reported to be overexpressed and associated with poor prognosis in several tumors. This study aimed to examine the expression of ROR1 and evaluate its prognostic significance in human lung ADC patients. In this present study, Western blot analysis and immunohistochemistry were performed to characterize expression of ROR1 protein in lung ADC patients. The results revealed that ROR1 protein expression was significantly higher in lung ADC tissues than that in their adjacent non-tumor tissues. Patients at advanced stages and those with positive lymph node metastasis expressed higher level of ROR1 (*P* < 0.001). Moreover, Chi-square test showed that ROR1 expression was correlated to gender (*P* = 0.028), the 7^th^ edition of the American Joint Committee on Cancer tumor-node-metastasis (AJCC TNM) staging system and lymph node metastasis (*P* < 0.001). Kaplan-Meier survival analysis indicated an association of high ROR1 expression with worse overall survival (OS) in lung ADC patients (*P* < 0.001). Multivariate COX regression analysis further confirmed that ROR1 is an independent prognostic predictor (*P* < 0.001, HR = 4.114, 95% CI: 2.513–6.375) for OS. Therefore, ROR1 expression significantly correlates with malignant attributes of lung ADC and it may serve as a novel prognostic marker in lung ADC patients.

Lung cancer, with increasing morbidity and mortality, is currently the most common cause of cancer-related death worldwide. Despite advances in early detection and standard treatment, lung cancer still has high recurrence rate (14%) and high mortality rate (27%) which is due to dimness of the pathogenesis of lung cancer. The 5-year survival rate of lung cancer is only 18%^1^. Non-small cell lung cancer (NSCLC) accounts for about 85% of all lung cancer and adenocarcinoma (ADC) is the most common histologic subtype^2^. Understanding the carcinogenic mechanisms of lung cancer may help to find a biomarker and further develop treatment approaches to improve the cure and survival rates of this cancer. Meanwhile, identifying high-quality cancer prognostic biomarkers is helpful to predict the outcome of different cancer patients and to avoid overtreatment to those patients at early stage^3^. Even Carcinoembryonic antigen (CEA) and CA125 have been identified as diagnostic protein biomarkers for lung cancer^4^, the sensitivity and specificity are not adequate for clinical use. Therefore, identification of novel and specific biomarkers with clinicopathological and prognostic significance is necessary for NSCLC management.

Although surgery, radiation therapy and chemotherapy can prolong the 5-year survival rate of NSCLC patients, especially for those at early-stages^5^, most NSCLC patients are diagnosed at advanced-stages due to asymptomatic characteristics and lack of effective biomarkers. For advanced NSCLC patients, platinum-based doublet chemotherapy remains the first-line therapy but the effectiveness is unsatisfactory mainly because of the non-responsiveness or resistance to chemotherapeutic drugs^6^. Recently, new treatments are developed based on novel molecular biomarkers^7^. For lung ADC patients carrying sensitizing epidermal growth factor receptor (EGFR) mutations, the small-molecule tyrosine kinase inhibitors (TKIs), gefitinib and erlotinib, have shown remarkable efficacy in first-line therapy^8^,^9^,^10^. Therapies against other oncogenic alterations including the Kirsten rat sarcoma viral oncogene homolog (KRAS)^11^, anaplastic lymphoma kinase (ALK)^12^ and ROS proto-oncogene 1^13^ are under investigation. Although such target therapies are effective, their benefit to OS is still limited, mainly due to the primary or acquired drug resistance^14^. Thus, identification of novel reliable markers and independent prognostic factors is important for improving therapeutic modalities and for prolonging the survival of NSCLC patients.

ROR1 is a transmembrane glycoprotein, belonging to the receptor tyrosine kinase (RTK) family. The structure of human ROR1 possesses one FZ (frizzled) domain, one Ig-like C2-type (immunoglobulin-like) domain, one kringle domain and one protein kinase domain. ROR1 plays a key role in normal embryonic and fetal development, and it is absent within most mature tissues^15^,^16^. Recently, some studies report that ROR1 expression is elevated in human leukemia and solid malignancies, such as breast, ovarian and pancreatic cancers^17^,^18^,^19^. Moreover, higher level of ROR1 is associated with more aggressiveness and poor prognosis in breast cancer and ovarian cancer, where ROR1 regulates expression of the genes involved in epithelial-mesenchymal transition (EMT)^20^,^21^. In lung cancer cell lines, ROR1 sustains a favorable balance between pro-survival PI3K-AKT and pro-apoptotic p38 signaling, and knockdown of ROR1 induces apoptosis in cancer cells^22^. Wnt5a, a ligand of ROR1, induces ROR1/ROR2 heterooligomerization to enhance leukemia chemotaxis and proliferation^23^. Nevertheless, there is no report about ROR1 expression and its relationship with clinical features of lung ADC patients.

In this study, we first analyzed the expression of ROR1 protein in lung ADC tissues by Western blot, then examined ROR1 expression in lung ADC with tissue microarray (TMA) by immunohistochemistry (IHC) analysis. Finally, we evaluated the correlation of ROR1 expression with clinicopathological features and survival of lung ADC patients.

## Results

### Elevated expression of ROR1 protein in lung ADC samples

**Figure 1:**
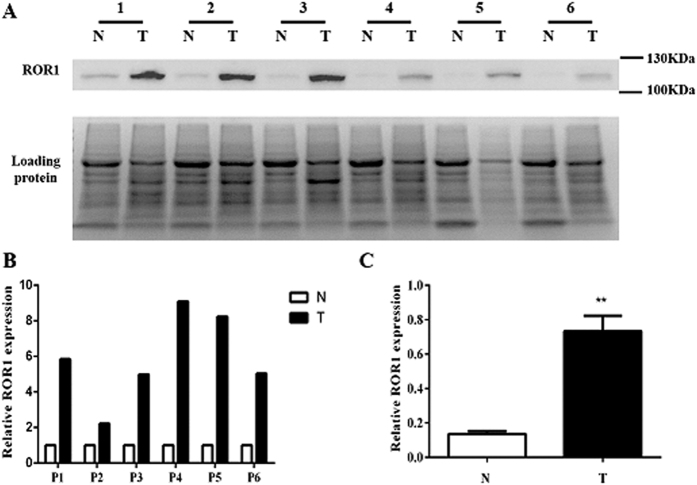
Analysis of ROR1 expression in lung ADC and their adjacent non-tumor tissue samples. (**A**) Western blot analysis showed that the lung ADC tissue samples (T) express high level of ROR1 protein while the adjacent non-tumor tissue samples (N) express little ROR1 protein. Total protein levels were used as loading control. The full-length gels of Western blot analysis are presented in Supplementary Figure S1. (**B**,**C**) The relative intensity of each panel was calculated by Image J and analyzed by paired t-test. Statistics results showed that the value of *P* < 0.001.

The expression of ROR1 protein was evaluated by Western blot analysis of lysates from lung ADC and their matched non-tumor tissue samples. The result revealed that lung ADC tissues expressed higher levels of ROR1 protein, whereas the matched non-tumor tissues expressed almost no ROR1 protein (Fig. 1A). The relative intensity of each panel was calculated by Image J and analyzed by paired *t*-test (Fig. 1B). When normalized to the total loading proteins, ROR1 expression in lung ADC samples was statistically much higher than that in the matched non-tumor tissues (*P* < 0.001) (Fig. 1C).

### Clinical features of lung ADC patients

To further validate elevated ROR1 expression status in lung ADC patients, tissue microarray containing 232 lung ADC cases was utilized to perform immunohistochemistry staining. The main clinicopathological characteristics of patients are shown in Table 1. The proportion of each group was almost equal by different clinical features. Overall, 109 female patients and 123 male patients, ranging in age from 25 years to 84 years (mean age of 61.0 years) were included in this study. According to the 7^th^ edition of the AJCC TNM staging system, 136 patients (58.6%) were at early stages (88 stage I and 48 stage II), 76 patients (32.8%) were at advanced stages (71 stage III and 5 stage IV), while the stages of remaining 20 patients (8.6%) were unknown. Meanwhile, according to the World Health Organization (WHO) pathological grade system, there were 11 (4.8%) at grade I, 169 (72.8%) at grade II and 52 (22.4%) at grade III cancers. The diameter of the tumor of 85 patients (36.6%) was ≤3 cm, while that of the remaining 147 patients (63.4%) was >3 cm. The most common histological type was pure ADC (179 cases), others including mixed-type ADC (27 cases), bronchiolo-alveolar carcinoma (16 cases), mucinous ADC (6 cases), papillary ADC (2 cases) and mucoepidermoid carcinoma (2 cases). There were 110 patients (47.4%) with positive lymph node metastasis, and 117 patients (50.4%) showed negative lymph node metastasis, while the status of remaining 5 patients (2.2%) was unknown. In all of the 232 cases, the survival information was available in 161 patients. The main clinicopathological characteristics of these patients are also shown in Table 1. Generally, the overall follow-up durations ranged from 1 to 121 months (mean time 40.8 months). Seventy-four patients were alive at the end of the follow-up and the overall survival (OS) rate was 46% in this study.

### Immunohistochemical analysis of ROR1 protein in lung ADC tissues

**Figure 2:**
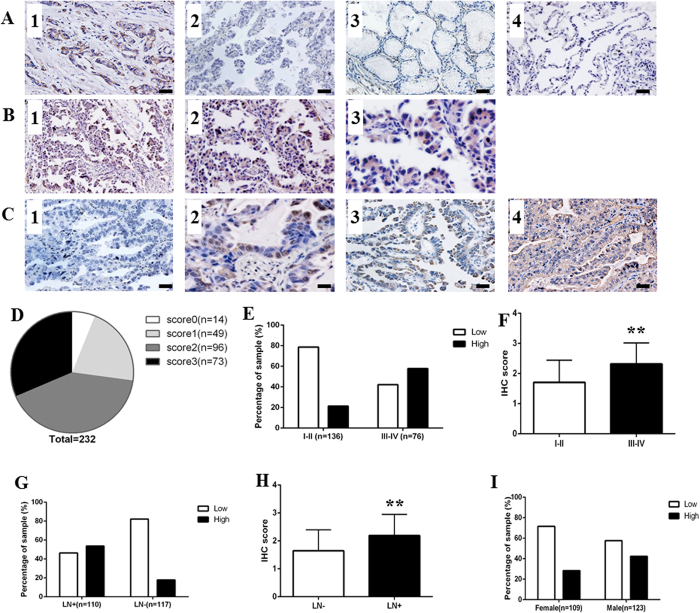
ROR1 expression in lung ADC tissue samples. (**A**) The IHC staining of positive and negative control revealed that ROR1 specifically expressed in cancer tissues. Breast cancer tissue stained with anti-ROR1 antibody was used as positive control (1). Negative controls included: lung ADC tissue incubated with PBS (2), nonspecific interstitial pneumonias (3) and adjacent non-tumor tissues (4) stained with anti-ROR1 antibody. (**B**) The IHC analysis of ROR1 expression in lung ADC tissues showed that ROR1 protein was mainly localized to the cell membrane and cytoplasm of lung ADC cells. Positive staining of ROR1 was shown in brown and the nucleus counterstained with hematoxylin was in blue. The magnification was ×100 in B1, ×200 in B2, ×400 in B3. (**C**) Different levels of ROR1 expression on TMA detected by IHC analysis. (1) Score 0 indicated that none or little cells express ROR1; (2) score 1 indicated that more than 25% of tumor cells have weak expression of ROR1; (3) score 2 indicated more than 50% of tumor cells have weak expression or more than 25% of tumor cells have moderate expression of ROR1 protein; (4) score 3 indicated more than 75% of tumor cells have moderate expression or more than 50% of tumor cells have strong expression of ROR1. Bar = 100 μm. (**D**) The pie chart represented the proportion of negative (score 0), weak (score 1), moderate (score 2) and strong staining (score 3) for ROR1 protein of TMA samples. (**E**) The proportion of low and high staining of ROR1 in lung ADC tissues of different stages was indicated in each bar (*P* < 0.001). (**F**) The scores of ROR1 in different stages were analyzed by two tail *t*-test. Statistical results showed that the value of *P* < 0.001. (**G**) The proportion of low and high staining of ROR1 in lung ADC tissues of different lymph node metastasis status was indicated in each bar (*P* < 0.001). (**H**) The scores of ROR1 in patients with different status of lymph node metastasis were analyzed by two tail *t*-test. Statistical results showed that the value of *P* < 0.001. (**I**) The proportion of low and high staining of ROR1 in lung ADC tissues of different gender was indicated in each bar (*P* = 0.023).

Because literatures showed that high level of ROR1 expression in breast cancer tissues, while their adjacent non-tumor tissues has little expression, so we first took advantage of tissue samples of breast cancer patients to validate the specificity of ROR1 antibody (Fig. 2A-1). Then we analyzed the expression of ROR1 protein in 232 lung ADC patients with TMA sections by IHC, and found that ROR1 protein was mainly localized to the cell membrane and cytoplasm of ADC cells, which is consistent with previous research (Fig. 2B)^15^. To minimize the bias of IHC scoring, we set up the scoring standard (Fig. 2C) and two independent researchers scored all of IHC staining samples. Considering the extremely high positive rate of ROR1 expression observed in this study, with the mean scored of 1.98 of all lung ADC tissue samples according to the 0–3 scoring system used in this study, we divided the lung ADC patients into two groups as follows: score ≤2 into the ROR1 low expression group and score >2 into the ROR1 high expression group. The positive rate of ROR1 expression in our study was 94% (218/232), with 21% (49/232) weak expression (score 1), 41% (96/232) moderate expression (score 2) and 31% (73/232) strong expression (score 3) (Fig. 2D). Additionally, our results indicated that the ROR1 expression is positively related to the 7^th^ edition of the AJCC TNM staging system. A total of 57.9% (44/76) patients at stage III-IV had high expression of ROR1 protein, but only 21.3% (29/136) patients at stage I-II exhibited high ROR1 expression (Fig. 2E). The scores of the patients at stage III-IV were significantly higher than those at stage I-II in two tail *t*-test (*P* < 0.001) (Fig. 2F). The similar result was found according to the lymph node metastasis status. In patients with positive lymph node metastasis, 53.6% (59/110) had high expression of ROR1, the percentage decreased to 17.9% in those patients with negative lymph node metastasis, and the *P* value (*P* < 0.001) of the difference about the scores in these two group reached the statistical significance (Fig. 2G,H). Noteworthy, although there was no significant difference observed about the scores in different genders (*P* = 0.18), higher ROR1 expression occurred more often in tumor tissue from male (42.3%) than that in female (28.4%) lung ADC patients (*P* = 0.028) (Fig. 2I).

### Relationship between ROR1 expression and clinicopathological parameters in lung ADC patients

The relationship between ROR1 protein expression and the clinicopathological parameters of lung ADC patients is analyzed by Chi-square test and presented in Table 2. The results revealed that high ROR1 expression was closely associated with advanced-stages (stage III and IV) (*P* < 0.001), positive lymph node metastasis (*P* < 0.001) and different genders (*P* = 0.028). There was no significant difference between ROR1 expression and other clinical parameters, including age, tumor diameter, pathological grade and histological type.

### Survival analysis

**Figure 3:**
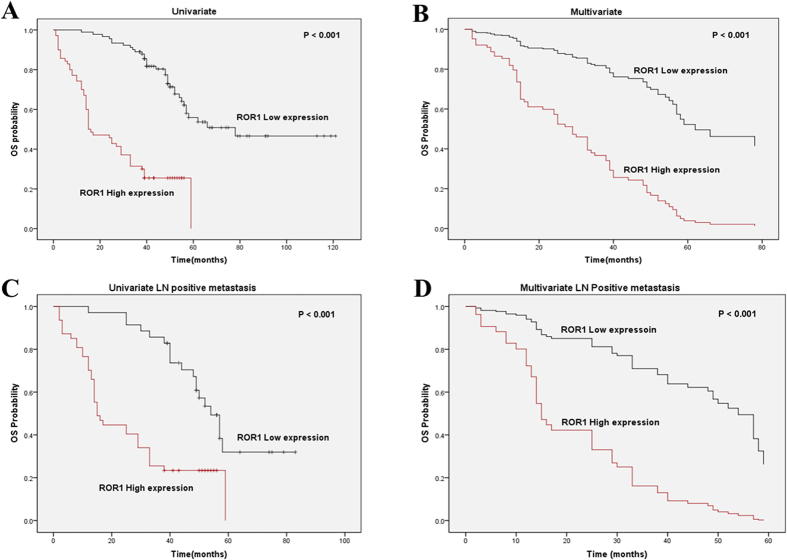
High expression of ROR1 is correlated with shorter OS in lung ADC patients and those with positive lymph node metastasis. (**A**) Kaplan-Meier survival analysis of ROR1 expression in 161 lung ADC patients split into two groups. Compared to the high ROR1 group, longer OS time was observed in the low ROR1 group. (**B**) Multivariate Cox regression survival analysis in 161 ADC patients split into two groups. ROR1 expression was determined to be an independent prognostic factor. (**C**) Kaplan-Meir survival analysis of ROR1 in 82 ADC patients with positive lymph node metastasis. Compared to the high ROR1 expression group, the low ROR1 expression group had better OS time. (**D**) Multivariate Cox regression survival analysis in 82 ADC patients with positive lymph node metastasis. ROR1 expression was an independent prognostic factor in these patients.

Of 232 patients, 161 patients were included in the survival analysis and the multivariate Cox proportional hazards model was applied to estimate the effect of ROR1 expression on survival. First, univariate survival analyses were employed to find the difference between lung ADC patients with different ROR1 expression levels. The log-rank test showed that in patients with different ROR1 expression levels, at different clinical stages and with different lymph node metastasis status, the median OS time were significant different (all *P* < 0.001) (Table 3). The Kaplan-Meier survival curves also showed that the lung ADC patients with higher ROR1 expression had a significantly unfavorable OS time (Fig. 3A). Meanwhile, the multivariate analysis indicated that ROR1 protein expression may serve as an independent prognostic factor for OS in lung ADC patients (HR = 4.114, *P* < 0.001) (Fig. 3B and Table 3), besides the 7^th^ AJCC TNM stage (HR = 2.879, *P* < 0.001). Moreover, we also stratified the lung ADC patients with positive lymph node metastasis status by ROR1 expression level, and revealed that higher ROR1 expression was also correlated with shorter OS (both *P* < 0.001) by both univariate and multivariate analysis (Fig. 3C,D). The results indicate that ROR1 might serve as an independent prognostic factor for lung ADC patients with positive lymph node metastasis.

## Discussion

Despite some progresses to treat lung ADC patients have been achieved in recent years, the 5-year survival rate remains unsatisfactory^24^. Finding more effective prognostic biomarkers in lung ADC patients is important for diagnosis and treatment, because the efficacy of different treatment strategies varies among different subgroups of patients^25^. For example, the lung ADC patients harboring *EGFR* mutations can benefit from the treatment of TKIs such as gefitinib and erlotinib^26^. Other potential biomarkers under investigation are mostly oncogene driver mutations, including the *ALK* gene translocation and *ROS1* gene rearrangements^12^,^13^. Furthermore, emerging cases of primary and secondary resistance to small-molecule target therapy indicate that new biomarkers are needed to specifically identify these patients^27^. Therefore, identifying a novel clinically-relevant prognostic biomarker for lung ADC is urgently needed.

ROR1 is an embryonic protein with three main structural domains, namely the extracellular immunoglobulin-like (Ig) domain, the cysteine-rich, frizzled-like domain and the kringle domain^15^. ROR1 has been shown to be critical for skeleton, cardiorespiratory and neurological development, but its expression is little in adult tissues^28^. Recent studies have revealed that ROR1 is highly expressed in several hematologic and solid malignancies such as CLL^29^, acute lymphocytic leukemia (ALL)^30^, renal cell carcinoma^31^, breast cancer^17^, melanoma^32^, and ovarian cancer^19^. The specific expression of ROR1 in cancer cells makes it a potential target for small-molecule TKIs and monoclonal antibodies (mAbs) for cancer treatment. The small-molecular compounds, KAN0439834 and IN0439365, have been shown to inhibit ROR1 kinase activity to exert the anticancer effect on CLL and pancreatic ADC cells^33^,^34^. Cirmtuzumab (UC-961), a first-in-class humanized anti-ROR1 mAb, had specific antitumor effect on CLL, breast cancer and pancreas ADC cancer without any off-target activity or toxicity in preclinical tests. UC-961 has been conducted to a Phase I study in patients with relapsed or refractory CLL^35^. Because of its tumor-specific expression and absence on normal mature cells, ROR1 could be a potential candidate for CAR (Chimeric antigen receptor) -T cell therapy. Hudecek *et al*. reported that T cells modified with an optimized ROR1-CAR have significant antitumor efficacy in a preclinical model *in vivo*, and the clinical study is about to be started^36^.

There were some reports about ROR1 expression in lung cancer, but most data were based on cellular and animal experiments. Zhang *et al*. reported that human lung cancer cell line A549 express surface ROR1^18^. NKX2-1(TITF1) has been reported to induce ROR1 expression and knockdown of ROR1 can inhibit lung ADC cell growth^16^. Only a few clinical studies have been reported with very small sample sizes. Analysis of 29 cases of lung ADC patients showed that 59% of them had strong expression of ROR1^18^. Karachaliou *et al*. analyzed the mRNA level of ROR1 in 27 NSCLC patients from the EURTAC trial (clinicaltrials.gov NCT00446225) who harbored EGFR T790M mutation, and showed that high ROR1 expression significantly limits progression-free survival (PFS) in the erlotinib-treated patients but not in the chemotherapy-treated patients^37^. Studies about ROR1 expression in human lung ADC patients and its relationship with clinical characters are limited especially by case numbers. So we examined the expression of ROR1 in 232 patients and did statistical analysis systematically in details trying to find out the clinical significance of ROR1 expression. First, Western blot analysis showed that ROR1 expression is much higher in lung ADC tissues than that in their adjacent non-tumor tissues, this was consistent with the previous reports^15^. Next, the IHC analysis revealed that ROR1 protein is mainly localized to the cell membrane and cytoplasm of lung ADC cells. More importantly, our data indicated that ROR1 expression level was associated with the 7^th^ edition of the AJCC TNM stage of lung ADC patients. Patients at advanced stages (III-IV) expressed higher level of ROR1 than those at early stages (I-II) (*P* < 0.001). Taken together, our results suggest that the expression level of ROR1 could be used to predict the clinical stages of lung ADC patients. On the other hand, the specific expression of ROR1 in lung ADC tissues made it a potential target for lung ADC therapy, thus we are currently developing novel small-molecule agents and monoclonal antibodies against ROR1 to treat lung ADC patients.

We also found a significant association between ROR1 expression and lymph node metastasis status in lung ADC patients. Higher expression of ROR1 was observed in patients with positive lymph node metastasis in our study (*P* < 0.001). Regarding the status of lymph node an important sign of metastasis in lung ADC patients, our findings indicated that ROR1 may be involved in the process of metastasis in lung ADC patients. Similar results have been observed in breast cancer where ROR1 protein level was higher in more aggressive subtypes^17^. A possible mechanism was suggested that ROR1 played a critical role in epithelial- mesenchymal transition (EMT) for cancer metastasis^21^. Silencing ROR1 expression by siRNA reduces the expression of EMT-associated proteins, such as SNAIL-1/2, ZEB1, CXCR4, and vimentin in breast cancer cell line MDA-MB-231. In ovarian cancer cells, miR-382 inhibits cell migration and invasion by targeting ROR1 to regulate EMT^20^. Accordingly, high expression of ROR1 correlates with metastasis and poor clinical outcome of malignant cancers. In the present study, we evaluated the association between ROR1 expression and survival with regard to clinicopathological factors in lung ADC patients for the first time. The Kaplan-Meier analysis demonstrated that the OS time of patients with higher ROR1 expression was shorter than that of patients with lower expression. In patients with positive lymph node metastasis, higher ROR1 expression was also significantly associated with poorer survival (*P* < 0.001). Multivariate analysis further confirmed that ROR1 protein expression can be used as an independent prognostic factor after adjusting for other clinicopathological factors. Though the specific functions and mechanisms of ROR1 in lung ADC need to be more exhaustively investigated, our data demonstrated that ROR1 could serve as an independent marker to predict the survival of lung ADC patients.

Notably, even there was no significant difference about the expression level of ROR1 in different genders in lung ADC patients, higher ROR1 expression occurred more often in male lung ADC patients than in female lung ADC patients with statistically significance in this study (*P* = 0.028). In male lung ADC patients, 42.3% expressed high level of ROR1 but in female patients it was only 28.4%. It is well known that gefitinib and erlotinib, the EGFR inhibitors, have a more favorable effect in non-smoking, female lung ADC patients. The percentage of lung ADC patients who harbored EGFR mutations was 51.5% in female and 35.7% in male patients (*P* < 0.01)^36^. This suggests that there might be interaction between ROR1 and the EGFR signaling pathways. Study with small samples showed that in erlotinib-treated patients with T790M mutations, high ROR1 expression limited the PFS time^37^. But the reason of this phenomena was unclear and warranted further investigation. We suspected the possible mechanism was that NKX2-1 and EGFR may be functionally interrelated with each other through the NKX2-1-mediated ROR1 induction in lung ADC cells. However, the specific mechanism of their interaction needs further investigation.

In summary, we investigated the clinicopathological relevance of ROR1 expression in a large cohort of lung ADC patients for the first time, and found that ROR1 is specifically expressed at higher levels in lung ADC tissue than that in adjacent non-tumor tissue. Moreover, ROR1 expression status in lung ADC patients correlated with different gender, the 7^th^ edition of the AJCC TNM stage and lymph node metastasis status. Patients at advanced stages or with positive lymph node metastasis had significant higher level of ROR1 expression. The Kaplan-Meier analysis indicated that patients with higher ROR1 expression had significant shorter OS, whereas those with lower ROR1 expression had longer OS. Multivariate analysis further confirmed that ROR1 is an independent prognostic factor for lung ADC patients. In conclusion, ROR1 expression is correlated with malignant attributes of lung ADC and may serve as a novel prognostic marker for lung ADC patients and provide a promising strategy for targeted therapy in lung ADC treatment.

## Methods

### Patient tissue samples

Human lung ADC and the adjacent non-tumor tissues were obtained from the Department of Thoracic Surgery, West China Hospital, Sichuan University, with informed written consent from each patient and used for Western blot analysis. Paraffin-embedded lung ADC and adjacent non-tumor tissue samples, nonspecific interstitial pneumonia and breast cancer tissue samples were collected from The Third People’s Hospital of Chengdu to be used as control in the IHC analysis. The clinical stage of the tumors was evaluated by experienced pathologist according to the 7^th^ edition of the AJCC TNM staging system. The original clinical data of the TMA including patient gender, age, tumor size, the 7^th^ edition AJCC TNM stage, tumor grade, histological type, lymph node metastasis status, OS time and survival status were obtained from Shanghai Outdo Biotech Co., Ltd. (SOBC). This study was approved by the ethics committee of the West China Hospital Affiliated to Sichuan University, and all experiments were carried out in accordance with approved guidelines of Sichuan University.

### Western blot analysis

Each lung ADC and the adjacent non-tumor tissues lysates were prepared and analyzed by Western blot as described^15^. Briefly, tissues were lysed with RIPA buffer (Beyotime, P0013B) containing protease inhibitor cocktail (Vazyme Biotech, E312-01-AA). The BCA protein assay kit (Beyotime, P0009) was used to measure the protein concentration. Then the proteins were separated on 8% SDS-polyacrylamide gels and electrically transferred onto PVDF membranes (Millipore, Billerica, MA, USA). Membranes were blocked for 1 h at room temperature prior to polyclonal rabbit anti-ROR1 antibody (Abcam, #ab135669) at 4 °C overnight. After being washed with TBST, membranes were incubated in horseradish peroxidase-conjugated anti-rabbit antibody (1:1000 dilutions, ZSGB-BIO, Beijing) for 1 h. After further being washed with TBST, membranes were developed using an enhanced chemiluminescence reagent and analyzed using an ImageQuant LAS4000 (GE Healthcare Life Sciences).

### Immunohistochemical analysis

The IHC staining was performed as previously described^16^. The tissue sections were rewarmed in the oven at 65 °C for 3 h and then deparaffinized in 100% xylene and dehydrated with graded alcohol. A pressure cooker heat-induced antigen retrieval method was used with 0.01 M citrate salt buffer (pH = 6.0, ZSGB-BIO, Beijing) for 3 mins at 95 °C. Then these tissue samples were naturally cooled to room temperature (RT) and incubated with 0.3% H_2_O_2_ for 10 mins to reduce the endogenous peroxidase activity. After being washed three times in Phosphate-Buffered Saline (PBS; pH 7.2–7.4, ZSGB-BIO, Beijing), these sections were blocked in the dark with goat serum for 15 mins, followed by incubation with the ROR1 antibody (Abcam, ab135669, 1:20) overnight at 4 °C in a wet box. After being rewarmed to 37 °C for 1 h, these slides were washed three times in PBS and incubated with horseradish peroxidase-conjugated anti-rabbit antibody (1:1000 dilutions, ZSGB-BIO, Beijing) for 15 mins at 37 °C. Negative controls were included by replacing the primary antibody with PBS. The reaction product was stained at RT with the prepared liquid DAB^+^ substrate-chromogen solution (Maixin Biotech. Co., Ltd. Fuzhou, China) for 30 seconds. After rinsing with distilled water, all of the sections were counterstained with hematoxylin. Two experienced pathologists without any knowledge of the clinicopathological information independently evaluated the result of the immunoreactivity. A semi-quantitatively scoring system (0–3) was used to evaluate the expression level of ROR1 as described previously^22^. The intensity of the staining was classified as negative, weak, moderate or strong. Staining intensity was scored as follows: 0 (negative), 1 (weakly positive), 2 (moderately positive), and 3 (strongly positive). The percentage of ROR1-positive cells was also scored according to 4 categories, where 1 was for 0–10%, 2 for 11–50%, 3 for 51–80%, and 4 for 81–100%. The product of the intensity and percentage scores was used as the final ROR1 staining score.

### Statistical analysis

Statistical analyses were performed by the SPSS 22.0 statistic software (IBM Corp. Armonk, NY, USA) or GraphPad Prism 6 software (GraphPad Software, Inc., La Jolla, CA). Briefly, the Chi-square test was performed to analyze the association between ROR1 expression and clinicopathological features. Two tail *t*-test was applied to analyze the ROR1 expression in patients with different clinical stages and lymph node status. In the univariate survival analyses, the difference of median overall survival (OS) time between groups of patients were analyzed by the log-rank test. The independent prognostic factors of OS were further identified by Multivariate Cox proportional hazards regression models. The hazard ratios (HRs) and 95% confidence intervals of the prognostic factors were calculated. Kaplan-Meier survival curves were constructed for survival analyses and differences were tested by the log-rank test. ROR1 expression was categorized as high or low using the median score. The results were considered statistically significant if *P* < 0.05.

## Additional Information

**How to cite this article**: Zheng, Y.-Z. *et al*. ROR1 is a novel prognostic biomarker in patients with lung adenocarcinoma. *Sci. Rep*. **6**, 36447; doi: 10.1038/srep36447 (2016).

**Publisher’s note:** Springer Nature remains neutral with regard to jurisdictional claims in published maps and institutional affiliations.

## Figures and Tables

**Table 1:** The basic clinical features of all 232 lung ADC patients and 161 cases with survival information.

Clinical Features	N_1_ = 232	N_2_ = 161
Age		
≤60	107(46.1)	75(46.6)
>60	123(53.0)	84(52.2)
Unknown	2(0.9)	2(1.2)
Gender		
Female	109(47.0)	75(46.6)
Male	123(53.0)	86(53.4)
Tumor Size		
≤3 cm	85(36.6)	63(39.1)
>3 cm	147(63.4)	98(60.9)
Pathological Grade^b^		
I	11(4.8)	11 (6.9)
II	169(72.8)	115 (71.4)
III	52(22.4)	35(21.7)
AJCC7 Stage^a^		
I	88(37.9)	53(32.9)
II	48(20.7)	32(19.9)
III	71(30.6)	55(34.2)
IV	5(2.2)	1(0.6)
Unknown	20(8.6)	20(12.4)
Lymph Node Metastasis		
Positive	110(47.4)	82(50.9)
Negative	117(50.4)	74(46.0)
Unknown	5(2.2)	5(3.1)
Tumor Type		
Pure Adenocarcinoma	179(77.2)	122(75.8)
Other	53(22.8)	39(24.2)

^a^Clinical stage was classified according to the 7^th^ edition of the American Joint Committee on Cancer tumor-node-metastasis (AJCC TNM) staging system. ^b^Pathological Grade was classified according to the criteria of the 2004 World Health Organization pathological grade system.

**Table 2:** The constituent ratio of different ROR1 expression level under different clinicopathologic variables in 232 lung ADC patients.

Clinicopathologic variables	n	ROR1		χ2	P-value
		Low	High		
All cases	232	149	83		
Age				0.261	0.609
≤60	107	67	40		
>60	123	81	42		
Unknown	2	1	1		
Gender				4.815	0.028*
Female	109	78	31		
Male	123	71	52		
Tumor Size				0.939	0.332
≤3 cm	85	58	27		
>3 cm	147	91	56		
Pathological Grade				0.039	0.843
I-II	180	115	65		
III	52	34	18		
AJCC7 Stage				28.882	<0.001*
I-II	136	107	29		
III-IV	76	32	44		
Unknown	20	10	10		
Lymph node metastasis				31.64	<0.001*
Positive	110	51	59		
Negative	117	96	21		
Unknown	5	2	3		
Histological Type				1.67	0.196
Pure Adenocarcinoma	179	111	68		
Other	53	38	15		

*P < 0.05.

**Table 3:** Univariate and multivariate analysis of prognostic factors in lung ADC patients for overall survival. HR = Hazard Ratio.

	Univariate analysis				Multivariate analysis				
	Median OS time	95% CI	Log-rank	p > | z |	HR	p > | z |	95% CI		
ROR1 expression			59.296	<0.001*	4.114	<0.001*	2.513–6.375		
Low	78	N/A**							
High	15	44.302–59.698							
Age			0.287	0.59					
≤60	52	44.708–69.292							
>60	49	37.011–60.989							
Gender			3.481	0.06					
Male	39	28.254–49.746							
Female	57	50.986–59.698							
Tumor size			5.889	0.02					
≤3 cm	78	N/A**							
>3 cm	48	44.302–59.698							
AJCC7 stage			36.922	<0.001*	2.879	<0.001*	1.805–4.593		
I-II	78	N/A**							
III-IV	29	17.113–40.887							
Pathological Grade			0.176	0.68					
I-II	54	45.908–62.092							
III	1.149	35.180–62.820							
LN			16.556	<0.001*					
Negative	78	N/A**							
Positive	38	29.339–46.661							
Histological Type			0.685	0.41					
Pure adenocarcinoma	54	47.889–60.111							
Other	48	26.379–59.698							

*P < 0.05. N/A**. The 95% CI cannot be calculated.
